# Ultrasound-Based Image Analysis for Predicting Carotid Artery Stenosis Risk: A Comprehensive Review of the Problem, Techniques, Datasets, and Future Directions

**DOI:** 10.3390/diagnostics13152614

**Published:** 2023-08-07

**Authors:** Najmath Ottakath, Somaya Al-Maadeed, Susu M. Zughaier, Omar Elharrouss, Hanadi Hassen Mohammed, Muhammad E. H. Chowdhury, Ahmed Bouridane

**Affiliations:** 1 Department of Computer Science and Engineering, Qatar University, Doha 2713, Qatar; s_alali@qu.edu.qa (S.A.-M.); elharrouss.omar@gmail.com (O.E.); hm1409611@qu.edu.qa (H.H.M.); 2 College of Medicine, Qatar University, Doha 2713, Qatar; szughaier@qu.edu.qa; 3 Department of Electrical and Electronics Engineering, Qatar University, Doha 2713, Qatar; mchowdhury@qu.edu.qa; 4 Centre for Data Analytics and Cybersecurity, University of Sharjah, Sharjah 27272, United Arab Emirates; abouridane@sharjah.ac.ae

**Keywords:** carotid artery stenosis risk, US, computer vision, deep learning, machine learning, segmentation, classification, plaque characterization

## Abstract

The carotid artery is a major blood vessel that supplies blood to the brain. Plaque buildup in the arteries can lead to cardiovascular diseases such as atherosclerosis, stroke, ruptured arteries, and even death. Both invasive and non-invasive methods are used to detect plaque buildup in the arteries, with ultrasound imaging being the first line of diagnosis. This paper presents a comprehensive review of the existing literature on ultrasound image analysis methods for detecting and characterizing plaque buildup in the carotid artery. The review includes an in-depth analysis of datasets; image segmentation techniques for the carotid artery plaque area, lumen area, and intima–media thickness (IMT); and plaque measurement, characterization, classification, and stenosis grading using deep learning and machine learning. Additionally, the paper provides an overview of the performance of these methods, including challenges in analysis, and future directions for research.

## 1. Introduction

The cardiovascular system is the major life-supporting system in the human body. Diseases of the cardiovascular system have emerged among the leading causes of death worldwide. According to WHO (World Health Organization) (https://www.who.int/news-room/fact-sheets/detail/cardiovascular-disease (accessed on 2 January 2022)) 2000–2019 statistics, an estimate of 17.9 million people died from cardiovascular diseases (CVDs), of which 38% were due to heart attack and stroke. The cause of this mostly lies in risky behaviors, such as alcohol and tobacco intake, poor diet, and physical inactivity. Early detection and management of the diseases can increase the life span and reduce the mortality rate due to CVDs.

The vast cardiovascular system consists of blood vessels transporting blood, which nourishes all the organs in the human body and ensures their functioning. Arteries and veins are the main constituents of the blood vessels that pump blood to and from the heart to all the organs. Any blockage of blood flow or malfunctioning of arteries or veins is a significant contributor to the improper functioning of the organs that they sustain. Coronary artery disease, carotid artery disease, and peripheral vascular disease are the most common forms of cardiovascular diseases. These diseases manifest due to the formation of atherosclerotic plaque in the arteries, which determines the progression of the disease. Ischemic stroke is one of the consequences of the plaque buildup in the carotid artery called carotid artery stenosis.

Carotid artery stenosis is an asymptomatic and persistent condition that is usually non-visible during routine checkups. The problem can be better managed if the stenosis is diagnosed early and the amount of plaque is quantified. Multiple imaging modalities are employed for this purpose. Diagnostic procedures performed for stroke assessment or evaluation of carotid artery stenosis involve magnetic resonance imaging (MRI), computed tomography (CT), electrocardiography (ECG), electroencephalography (EEG), and ultrasound imaging (US). In addition to imaging-based analysis, laboratory tests for coagulation status and heart monitoring are also performed. However, these are performed at the onset of stroke or during a routine examination with known symptoms of ischemic stroke due to CVDs.

**Figure 1:**
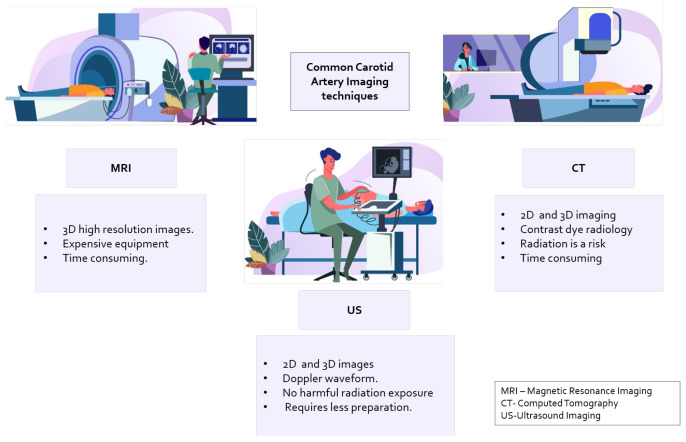
The different modalities for imaging the carotid artery for stenosis risk diagnosis.

The common carotid artery is located internally on both sides of the neck [1]. Plaque buildup is gradual, occurs in different stages, and is spatially distributed on the arterial walls. The arteries consist of soft tissue structures that enable them to be imaged with different techniques, termed “modalities” in [2], such as computerized tomography (CT), magnetic resonance imaging (MRI), and ultrasound imaging (US) [3]. Common imaging devices for imaging are shown in Figure 1.

Image analysis of images generated with these modalities can improve diagnosis and aid in clinical decision-making. Tissues, organs, and blood vessels are visible in different spectra, such as X-ray gray scale, Doppler spectrum plot, etc. Trained operators or radiologists are typically employed to grade the plaque to determine the level of stenosis in the subjects [4]. However, human error and image distortion due to factors like occlusion can cause misinterpretation. This leads to the need for an automated technique to aid in diagnosis.

From image processing and pattern recognition techniques to machine learning and deep learning algorithms that consider it a computer vision problem, medical image analysis has made significant progress. In the context of carotid artery images produced with CT scans, MRI, and ultrasound images, there has been accelerated development [5] in the analysis, segmentation, and grading of stenoses.

In a traditional approach, a radiologist marks the thickness of the intima–media in a region, marks the plaque area on the carotid artery, and grades the stenosis based on a grading standard. This introduces chances for human errors and is highly dependent on the radiologist’s skills. For large sets of data that may need a bulk diagnosis, an automated approach that is based on artificial intelligence, in its optimal configuration, could outperform human inference and reduce diagnosis errors.

In this light, robust analysis of the images generated with imaging tools is required. To this end, this review presents carotid artery stenosis indicators and their formation. Then, the imaging techniques and the devices used for this purpose are briefly introduced. The analysis of the images acquired from the devices is presented, along with the dataset used, analysis techniques, and grading of plaque presence, with a focus on ultrasound imaging. The challenges, dataset used, and its performance are presented for real-time applications, and the point forward is proposed. Several reviews have addressed several aspects of carotid artery imaging and analysis such as [6,7] in several domains, but here, we present a comprehensive overview of the problem.

In brief, an overview of the problem, the formation of the carotid artery, the dynamics of the stenotic plaque, the diagnosis techniques, the imaging techniques (including most common techniques and their flaws), and the analysis of the images generated to identify carotid artery risks. Furthermore, the article elaborates on the most prominent image analysis techniques, the datasets used, and the automated segmentation, classification, and plaque characterization techniques. This comprehensive analysis leads to an overview of the problems and solutions presented in the state of the art for carotid artery risk stratification. The main contributions of this article lie in the analysis of the problem, the presented solutions, their limitations and challenges, and some prospects for the analysis of carotid artery risk stenosis prediction.

This review will further concentrate on the first line of evaluation for carotid artery stenosis risk prediction: ultrasound images. Existing reviews in the field of US image analysis from Molinari et al. [8] and Mao et al. [4] present existing segmentation methods through feature extraction and classification. Most advanced techniques like deep learning were later surveyed for segmentation in a review published in 2021 [6]. However, the recent approaches and the latest results are required to be presented and the effectiveness of each analyzed. The article in Section 5 summarizes the machine learning and deep learning techniques used in carotid artery classification, segmentation, and plaque characterization.

The contributions of this paper are:

- The problem is stated in detail with respect to the localization of the arteries, high risk areas and the anatomy of risk development.
- Automated medical image analysis techniques in existing literature including a brief overview of segmentation, classification and plaque characterization methods from year 2016 to the present using ultrasound images.
- A brief analysis of the methodology used in particular, machine learning and deep learning methods.
- A brief overview of datasets from the literature obtained from ultrasound images either manually or automatically with its demographics as well as artifacts that may arise due to other factors such as noise or movement.
- A discussion on the methodology used as well as the challenges and the future directions.

With respect to image analysis, the review presented identifies that a myriad of transfer learning techniques have been utilized. While segmentation and object detection have been performed for localization, classification of the extracted region of interest is performed consequently. Further, plaque characterization is performed by identifying the type of plaque. Each method is either performed as an individual study or as a pipeline for classification. Other than classification, grading the stenosis or plaque buildup is performed by measuring the localized area using geometrical calculations. The current state of the art shows prominence in deep learning methods where feature engineering is not required; rather, layers of the models extract high level to low level features.

Examining the state of the art in detail, the anatomy of the carotid artery, which is where and how the risk of stenosis develops, is introduced in the succeeding section. In the section after, common visualization tools used for imaging the carotid artery are identified. Then, datasets acquired from each imaging tool are surveyed, with the most common tool as the point of focus (ultrasound imaging) US. Moving forward, a discussion on the latest state of the art in automation techniques that is deep learning, and machine learning for US analysis is performed. A discussion is performed based on image analysis, challenges, and future directions in research in this area. The final section concludes the survey with a brief outlook on the findings.

The following section consists of brief background into the carotid artery anatomy and its dynamics, the intima-media, lumen, and its role in ischemic stroke determining the need to localize the carotid artery for diagnosis.

## 2. Background

**Figure 2:**
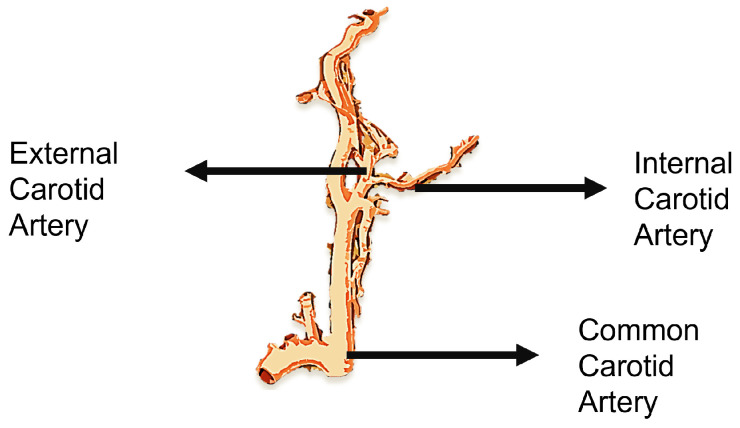
Common carotid artery diverging to the internal carotid artery and external carotid artery.

The heart is the main blood-pumping organ that regulates a complex circulatory system that allows blood to circulate throughout the body, forming the cardiovascular system. The cardiovascular system, which supplies blood to one of the major organs, the brain, is one of the most crucial life-sustaining systems. The brain requires a constant supply of oxygen provided via the bloodstream to function. The carotid arteries are quintessential blood vessels in the neck that carry oxygen and nutrients to the brain, neck, and face. The common carotid artery, the artery that leads to the brain, diverges into internal and external carotid arteries, as seen in Figure 2. The internal carotid artery (ICA) is responsible for supplying blood to the brain, and the external carotid artery (ECA) is responsible for blood flow to the neck.

A severe disruption in the blood flow in the carotid artery may have fatal consequences. Furthermore, due to the prominence of its position in the cardiovascular system, connecting the heart to the brain, it affects a person’s cardiovascular health.

These consequences are caused by plaque that can build up in the arterial wall and harden over time, thickening the artery wall and narrowing the oxygenated blood supply line, a condition known as stenosis [9]. Plaque is composed of waxy minerals such as calcium, fat, cholesterol, and other substances that dissolve in the circulatory system, also called atheroma formation. This was corroborated in several research areas related to ultrasound image analysis of stroke patients, coagulation studies, angiography using MRI and CT scans, and in the latest molecular biology, all of which prove that rupture of plaque is a key mechanism for cerebrovascular events [10].

**Figure 3:**
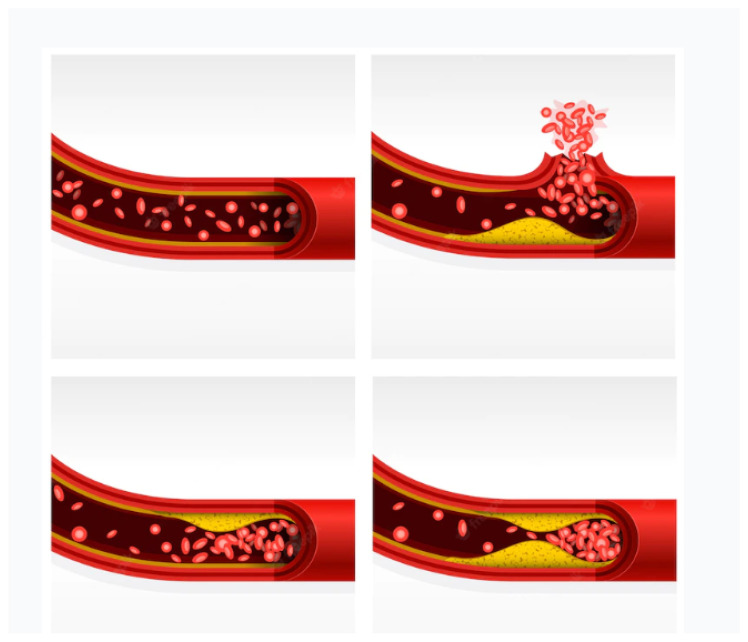
Stages of stenosis from normal flow to plaque deposit and rupture.

Due to the build up of plaque, the bifurcation site of the common carotid artery can produce turbulent blood flow if swollen. The atheroma/plaque is most likely to be formed at this site, where the internal carotid artery is at the most risk. This leads to neurological symptoms that are headache, dizziness, weakness in muscles, and, at major, if blood flow is completely blocked, cerebral ischemia or stroke [11]. Figure 3 is an illustration of different stages of plaque formation in an artery leading to stenosis and then rupture. Plaque deposits are viewed during imaging and the estimation of how much they occlude the blood vessel and obstruct blood flow or if any ulceration in the artery is identified.

The atheroma formed quantifies the stenosis, which can determine the risk of CVDs. According to the European Carotid Surgery Trial (ECST) in [12], CVD risk is classified based on the degree of stenosis. Patients with 30% to 70% stenosis are recommended for medicinal treatment, and the risk versus the benefit of a surgical intervention to improve the condition is based on the condition of the patient. However, with a 70–90% stenosis where there is a high risk of stroke or post-stroke, patients are recommended for blood clotting medications as well as surgical procedures if required.

The features that indicate stenosis that can be leveraged for diagnosis involve

- The internal carotid artery/common peak systolic velocity (ICA PSV) and internal carotid/common carotid end-systolic velocity ratio.
- Hemodynamics is altered due to certain stenosis degrees usually increasing the peak systolic velocity.
- Plaque quantity and characterization. The estimate of the plaque defines the amount of carotid stenosis in the arteries as a higher amount of plaque produces a higher risk of stroke.

**Figure 4:**
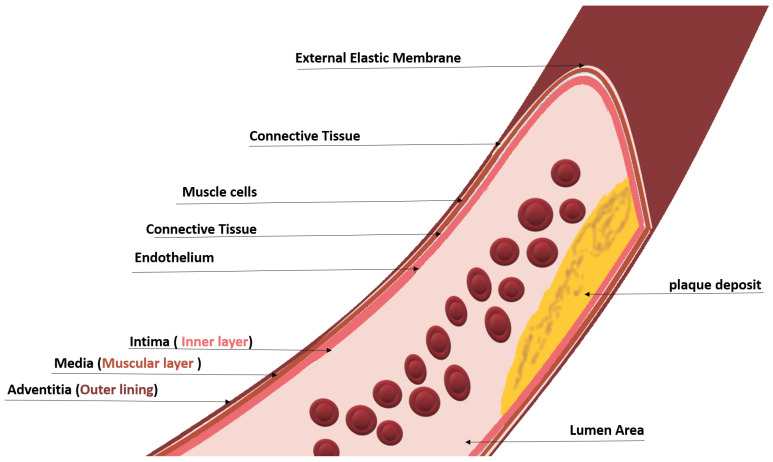
Anatomy of the inner layers of the carotid artery.

To measure the amount of stenosis, invasive and non-invasive techniques are employed. The carotid artery is subjected to imaging techniques. Cross-sectional and longitudinal images are generated from imaging modalities such as MRI, CTA, pulse wave, and ultrasound. The first line of diagnosis is typically ultrasound imaging with an echocardiogram to determine the presence of plaque as an initial analysis. The principle behind identifying the degree of stenosis through imaging lies in the anatomy of the carotid artery, which is shown in Figure 4; different layers of the carotid artery are shown depicting the intima–media–adventitia, where plaque deposits and causes stenosis [13].

As can be seen in Figure 3 and Figure 4, the plaque deposits on the inner layer (lumen) of the artery. Carotid plaque protrudes into the lumen, which is composed of calcified deposits (hard plaque), non-calcified deposits (soft plaque), or a combination of calcified and non-calcified material. The grading is performed by measuring the intima–media thickness (IMT). The vessel gap (lumen area) is narrowed with plaque buildup, and so the measure of the lumen area with and without the intima–media can be used to determine the stage of stenosis. In the North American Symptomatic Carotid Endarterectomy Trial (NASCET) [14], the intima–media thickness was found to be increased for patients who had undergone a stroke or myocardial infraction and had a history of CVD by 6–12% compared to those without CVDs. Patients with other comorbidities that comprise diabetes, smoking, and hypertension have also been found to have a thicker IMT of 5–12% [15,16,17].

According to the American Heart Association committee, in a normal arterial intima in the straight part of the common carotid arteries, the distal 1 cm is the normal thickness and comprises a single layer of attenuated endothelial cells over a sub-endothelial connective tissue giving a thickness of approximately 0.02 mm. In a normal CCA, the IMT is primarily the tunica media [18]. Larger measurements may indicate a plaque deposit and a higher risk level of atherosclerosis. Kassam et al., in [19], performed a Doppler waveform analysis. The result of the evaluation was that for 52% stenosis, the waveform generated distally to 8 cm past the stenosis was preserved.

The process of identifying the degree of carotid artery stenosis is termed grading. The recommendation for grading by [20] is as tabulated in Table 1. The ICA PSV (Internal Carotid Artery Peak Systolic Value) is the blood flow velocity in the systolic cycle at m/s. The ICA/CCA PSV is the ratio of the internal carotid artery PSV to that of the main common carotid artery PSV. The ICA EDV is the internal carotid artery end-diastolic velocity, which is the velocity of blood flow in the ICA at the end of the diastolic cycle in m/s.

The significance of stating the background allows proper design of a solution for carotid artery stenosis risk prediction. Stenoses, aneurysms, thromboses, and diseases caused by atherosclerotic plaques or congenital abnormalities formed in the carotid artery have a plaque property associated with them. Imaging the carotid artery can enhance our vision of the disease, if any, or rule it out; it can also determine how much risk a person is at in terms of the progression of plaque by grading it. Analyzing these diseases requires a good understanding of how the human cardio-vascular system works when blood flow is blocked or blood pressure is increased due to plaque development and the narrowing of arteries. Blood flow analysis [21,22,23], through sonograms, visualizing the artery through photoelectric plethysmography [24], auditory analysis [25] were initial approaches for assisting diagnosis. Further, CTA, MRA [26], and US [27] imaging were introduced, which further enhanced the visualization process [28].

**Figure 5:**
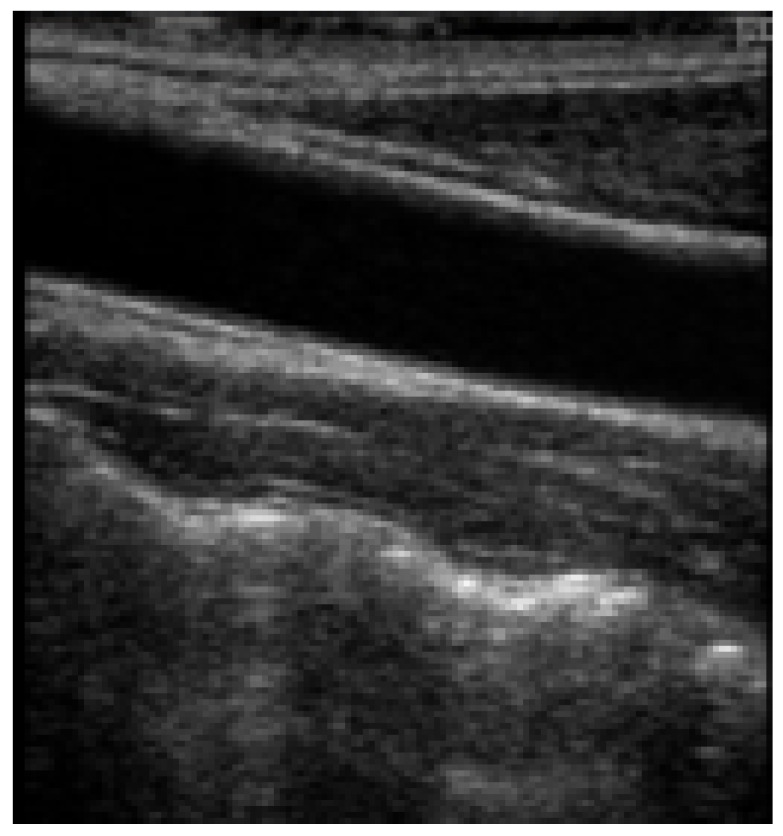
B-mode ultrasound image from publicly available CUBS dataset [31]. Accessed by CC by NC 4.0 license.

In 1986, Pignoli et al. [29] introduced the first computerized method for IMT segmentation of images acquired from ultrasound images. Later in 1990, the edge detection technique was implemented by Touboul et al. for IMT segmentation, which produced a good correlation compared to the ground truth [6]. Prior to that in 1988, advanced analysis techniques were performed on several imaging tools consisting of Magnetic Resonance Imaging, Computed Tomography, pulse wave, Doppler ultrasound, etc., for segmentation of the region of plaque and grading stenosis [3]. Furthermore, empirical examinations on in vivo and in vitro subjects, including both human and animal studies, have shown that the deposit of atherosclerotic plaque results in the dilation of the arterial wall and does not change the luminal size of the artery, allowing the arterial wall to be evaluated using these modalities [29]. Other than image processing, spectral analysis is conducted on Doppler US signals [30]. From then on, several imaging techniques such as MRI, CTA, DSA, Doppler waveform, B-mode ultrasound, etc., were widely implemented and analyzed. Figure 5, shows a B-mode ultrasound-generated image of the carotid artery region from the dataset published for public usage by Meiburger et al. in [31].

Table 2 is a list of references from the state of the art that determines the efficacy of different imaging modalities for carotid artery stenosis evaluation. The accuracy of annotation for carotid artery stenosis risk identification and the correlation reference is used to evaluate and validate the imaging technique. The sensitivity, specificity, and accuracy of statistical methods were used as metrics to evaluate the correlation. Several expert observers were used in the state-of-the-art papers to agree or disagree on a given label or conclusion by which the imaging data were observed and sometimes correlated with retrospective data.

The following section describes the latest methodologies employed for automated analysis, which include the process of segmentation, object detection, and classification of significant areas in the carotid artery for the purpose of stenosis grading and classification using computer vision algorithms that include artificial intelligence, its subsets, deep learning, and machine learning.

## 3. Methodology

### 3.1. Ultrasound Imaging

A medical imaging and diagnostic tool that is cheaper and lighter than the alternative is ultrasound. Apart from that, the method of imaging is non-invasive [35]. Its use is ubiquitous in terms of application and region of interest. With the advancement of tools and knowledge-building algorithms, the complexity of operations and inference has improved. There are several types of US, with the basic being an ultrasound pulse wave propagating through the body where the boundary between tissues reflects the sound energy that is received and analyzed. There are several types of ultrasound imaging based on the wavelength, scattering, and type of transducers, such as A-mode, B-mode, M-mode, TM-mode, and D-mode. Doppler US, pulse wave Doppler, continuous Doppler, and duplex Doppler are types of D-mode US.

The ultrasound device consists of three components: the transducer, or the probe, which contains the receiving and transmitting equipment; the probe is connected to a machine that analyzes the received signal and plots the image for viewing; and the probe is usually placed at an angle to the location of the carotid artery, that is, the left and right sides of the neck. Typically, in a B-mode ultrasound device, the probe is placed at 60 degrees from the ROI, whereas the Doppler US probe is placed at 90 degrees. The types of ultrasound imaging include B-mode imaging, Doppler sound imaging, continuous-wave Doppler ultrasound, and Duplex Ultrasound.

In B-mode imaging, anatomical structures are displayed on a 2D plane in ultrasound scanning using echography or B-mode US [36]. The reflection and diffusion of US waves are captured by the transducer, which transmits the waves. The captured signals are displayed based on the US amplitude of the signal in a gray-scale format with varying black and white intensities.

In Doppler Ultrasound, carotid artery blood flow in terms of linearity and velocity per volume is measured using Doppler US. Doppler US is used for assessing the degree of stenosis in the artery and obturations, and it identifies pathways for collateral circulation.

In continuous-wave Doppler ultrasound images, the extracranial arteries, or the arteries transporting blood to the base of the skull, are subjected to examination using a continuous-wave Doppler ultrasound. The frequency of ultrasound used for this method ranges from 2 to 20 MHz. For deeper vessels, lower frequencies are set.

A combination of the pulse wave and color Doppler is duplex ultrasound. Typically, an initial diagnosis of the carotid artery is performed with duplex ultrasound. It produces no radiation and does not require a contrast dye, resulting in a painless and safe diagnosis. In addition, it is a non-invasive analysis method. Duplex US uses sound waves to assess the blood flow in the arteries. The blood flow velocity measured can estimate the diameter of the blood vessel, thereby determining the amount of obstruction caused [2].

Although marginally clear images can be visually represented using ultrasound imaging techniques, there are some artifacts due to motion, noise, occlusion [37], acoustic shadow, mirror image mimicking artifacts [38] and dose-dependent artifact [39]; some of them have a significant effect on representing stenotic vessels.

The lumen area, intima–media thickness, and adventitia are identified to evaluate the risk of stenosis or quantify and grade it. However, images may contain artifacts where the effects of blur and signal loss in certain regions may lead to misdiagnosis. Knowing an artifact’s typical location and appearance helps avoid misinterpretations or misdiagnosis [40,41]. Image denoising is a method of artifact removal from acquired images. Region of interest segmentation and plaque localization are other pre-processing steps that can be employed to improve performance.

### 3.2. Image Pre-Processing

For better analysis, an enhanced image is required. Noise caused by several factors mentioned as an artifact in the previous sections requires the image to be pre-processed for better inference. Several types of noise can be found in images, such as Gaussian noise, impulse noise, photon noise, and speckle noise. In the context of MRI Images, de-noising is performed when artifacts are found in the acquired image [42]. Calcium deposits, and device settings, are some common problems in US images. Segmentation tasks may be misaligned, and ROI selection can be incorrect, leading to unreliability of information gained. Delsanto et al. in [43], as an example, orchestrated an advanced CULEX method due to the presence of fibrous and hard plaques that were not identified in the initial study. Several statistical and filtering methods can be used to perform de-noising in carotid artery images, some of which are listed in Table 3 with the majority of them pertaining to B-mode US.

Furthermore, the comparative analysis in [51] presents the different types of US filtering techniques, including statistical filtering, median filtering, geometric filtering, and homomorphic filtering, each of which was measured on its image enhancement ability through statistical analysis, texture features, a KNN Classifier, and optical perception evaluation. The conclusion entailed the use of local statistics (lsmv and Isminsc) and geometric filtering (gf4d) for pre-processing. Other forms of filtering used hybrid filters, such as in [52], which applied the Levy shrink method and gamma distribution analysis, and knowledge-based segmentation [27], which is a larger field of study. Authors in [34] identified that a high-frequency filter cannot be used as a pre-processing tool to remove artifacts as the Doppler signal components of a diseased person are close to 0 Hz. The Snakes algorithm, dynamic programming, first-order absolute moment, anisotropic Gaussian derivative filters, and first-order Gaussian filters are among the available pre-processing techniques for Doppler US images [53,54,55,56,57,58,59,60,61,62,63,64]. With each modality in imaging, there are different noise characteristics, and thus customized filters are required based on the image dataset available. An image correlation study like the chi-squared test, which was adopted in [34], to quantify the significance of the image enhancement approach to improve risk classification. Furthermore, with the popularity of machine learning and deep learning techniques, automated methods have been introduced that can be used to de-speckle and de-noise images. This area requires further study in terms of carotid artery image pre-processing. Segmentation of the plaque before characterization or classification is widely performed in the state of the art, which is mentioned in Section 6.

Apart from image enhancement, Doppler US produces signal plots, where the window height is determined by quantifying the difference between the maximum and minimum frequencies. Vessel wall movement artifacts can cause minimum frequency measurements to be faulty. To reduce this artifact, the first moment of the instantaneous amplitude spectrum is chosen in [34]. Table 3, is a depiction of the most recent literature on pre-processing techniques applied to images. In addition, the Atheromatic 2.0 [65] an automated plaque region extraction tool was used in the state of the art literature for classification of plaque types and characterization of the plaque, such as in Sanagala et al. in [47] and Skandha et al. in [66]. Further review is performed in Section 6.

### 3.3. Analysis Approaches

Automated analysis of the carotid artery through images and signal processing has become possible through the introduction of advanced algorithms like machine learning. Typically, the images are acquired by a radiologist. During image acquisition, the radiologist or operator controls many parameters, such as frequency, focus, zoom, probe orientation, and pressure on the tissue being examined. This leads to multiple variations in the data, leading to the requirement of a robust technique to analyze each variation. Several algorithms have been proposed, of which the latest is deep learning, which primarily requires large sets of data. Early image analysis research focused on multi-step filtering and feature extraction methods.

With the introduction of artificial intelligence, deep learning and machine learning have revolutionized data-driven medical image analysis. Several multi-step algorithmic approaches have been replaced by a machine learning model that can infer on the data based on a given input feature. The following subsection is a brief introduction to these approaches used in carotid artery stenosis risk prediction tasks.

#### 3.3.1. Machine Learning

Machine learning is a subset of artificial intelligence. Features are engineered and fed to a learned model to predict. The data given are of high importance. The existing data in the context of carotid artery ultrasound images are described in Table 4. The data are represented as features, and these features are trained on a classifier, predictor, or regressor algorithm. Feature engineering approaches are used to identify the most appropriate feature to represent the given data. The goal of a learner (classifier, regressor, or predictor) is to generalize from the learned data, which is a set of historic data with broad representation. Given unseen data, the learner should be able to perform accurately. The learner has to create a model from the given sample space to be able to distinguish the components in it and decide on new data. Several techniques are presented for machine learning, among which are supervised approaches with labeled data, unsupervised approaches, and reinforcement learning approaches.

In the context of medical image analysis, the anatomical structures are required to be identified, and image features are extracted using different image filters. The choice of different image filters which are designed manually, forms part of the feature engineering process [80].

#### 3.3.2. Deep Learning

Deep learning has made a huge advancement in classifying and quantifying patterns for medical image analysis [80]. Deep learning provides a hierarchical feature representation that requires no feature engineering but rather perform an automated feature extraction. Computer vision approaches in particular, which include image segmentation, image fusion, image annotation, and microscopic image analysis, have had a breakthrough due to deep learning.

Deep learning input data are usually represented in two modes: in vector form, a non-structured data, or in a 2D or 3D shape, which is structured and uses convolutional neural networks. With images, convolutional neural networks are constructed, which have made significant contributions to medical image analysis.

A basic deep network consists of a feed-forward neural network, which consists of an input layer and an output layer. A single layer of this is called a perceptron. In order to attain improved accuracy, a multi-layer perceptron is applied with multiple hidden layers. The neighboring units in each layer are fully connected. Non-linear activation functions are employed to improve accuracy and provide a hierarchical representation of features. Gradient descent and back-propagation are employed in the feed-forward network to update the weights.

A better representation of an object is obtained by adding more layers and training with larger datasets achieving better accuracy based on the case. Convolutional neural networks detect local features at specific locations determined by the filter or learnable kernels, which are updated randomly during the feed-forward process to achieve better representation.

Large models trained on large datasets are typically used as pretrained models with the classification layer detached for fine-tuning for a similar class termed as transfer learning. Some of these large models are used as feature extractor backbones for several state-of-the-art medical image analysis applications, such as the Unet [81] and Deeplab [82] for segmentation purposes.

Advantages of convolutional approaches include sparse interactions, weight sharing, and translation equivariance. Self-attention mechanisms enabled focus on semantic regions in CNN models where self-attentive feature maps are fed as input. Thus, localizations of clinical feature regions are significantly represented [83].

To overcome the shortcomings of convolutional networks, that is, their fixed operation and inability to model long-range interactions, convolutional-free approaches are sought after. In addition to this, non-local correlations of objects in images and long-term dependencies in images are neglected in CNNs [84]. Inspired by the performance of transformers, a sequence-to-sequence model in natural language processing, Vision transformers were proposed in Doso- vitskiy et al. From then on, several variations of this approach have been used for computer vision tasks such as segmentation [85] and classification [86]. Vision transformers convert the input image into a set of image patches tagged with an image sequence, capturing global features and dependencies in the image itself.

## 4. Dataset

The success of machine learning and deep learning models depends on reliable datasets marked and labeled by radiologists and physicians. Fair and unbiased data are a requirement for generalization across a larger population in terms of medical images. Due to the existence of several attributes tagged to medical images, such as age, gender, race, ethnicity, and other illnesses, unbiased data are ever more required for an automated system that can scale across several countries. Thus, the following section presents the dataset distribution across continents as well as the methods used to attain the dataset.

Datasets are acquired from medical devices tested on patients who are willing to take assessments with permission acquired through an institution’s or country’s review board on ethics of data collection and patient data handling. In the context of ultrasound images, the images acquired are acquired through ultrasound devices placed on the carotid artery area, that is, the neck. The probe is either placed lengthwise to capture the ultrasound signal, producing longitudinal images, or breadthwise textcolorblueto capture a cross-sectional view of the artery, named transverse view images. Each institution that has produced the dataset has used different types of devices and different sets of population as described in the following section.

### 4.1. Doppler Datasets

Dataset1 [34]: The Doppler dataset presented in [34] consists of a training set of diseased and normal arteries. This is one of the first datasets for automatic segmentation of the carotid artery. The diseases are assessed separately by contrast arteriography by a radiologist. If the vessel diameter was Vdiam, the stenosis estimate measure was performed by

(1) $$PercentageStenosis=100\times 1-\frac{unoccludedVdiam}{totalVdiam}$$

The profile of the patients ranges from 38 to 82 years for the diseased arteries, with a mean age of 61.7, and the normal arteries are of asymptomatic patients without bruits, with an age range of 21 to 43 years and a mean age of 28.5. These data are collected from four sites of each carotid artery system: proximal (approx. 3 cm proximal to the bulb) and distal (approx. 1 cm proximal to the bulb) of the CCA. Feature extraction algorithms are used in [34]. A feature selection process is performed with high-weighted features selected and residual correlation is eliminated. These data were tested with a hyperplane solution and a multivariate distance criterion. The algorithms used are the plane, least discriminant analysis, Pietrantonio–Jurs, and k-NN, where 94–100% accuracy was achieved for this dataset.

PICMUS Challenge-Doppler: The PICMUS dataset was created as part of a challenging dataset for medical image analysis. It contains plane-wave ultrasound data. The challenge was issued to outperform the state of the art in the detection of carotid arteries for this dataset. The ultrasound toolbox [87] can be used to beamform and display the data.

### 4.2. B-Mode Datasets

The following is a detailed description of the datasets used in the state of the art. The results and analysis of them are presented in the Section 5.

**Figure 6:**
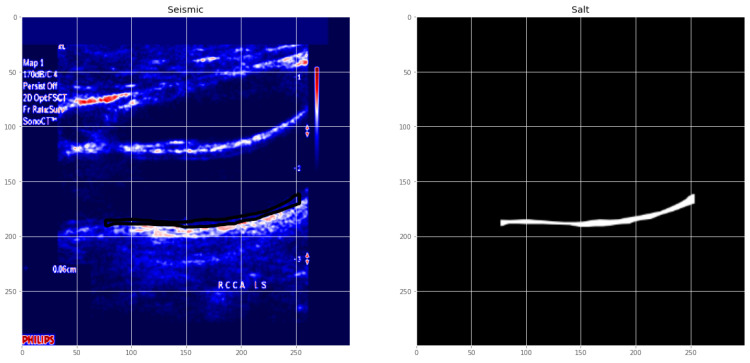
A sample of ultrasound B-mode dataset annotated for its intima–media layer mask.

CUBS dataset: The dataset consists of 1088 patients from two different institutions. The correlation parameters were measured by skilled analysts who segmented the US images. The comparison with automated segmentation was positive. This dataset was annotated for intima–media thickness, and ground truth was provided by manual as well as automatic segmentation methods. The first version of the dataset consists of 2716 images, and the second version by the same authors consists of 500 images of the carotid artery. These datasets are publicly available in [31,76]. Figure 6 is a sample of the dataset with the intima–media mask.

CULEX-B mode: A Philips ATL HDI 5000 ultrasound scanner was used for acquiring the US B-mode images. A 5mm linear probe was used at an operating frequency of 12 MHz. Then, 120 images from 31 subjects with an age range of 41–89 were used, of which 22 were healthy and 9 were had intima–media thickening. A neurologist, a cardiologist, and two radiology technicians manually segmented the images [8].

Japan Database (JDB): A B-mode US was used to acquire 404 images from the left and right CCAs. The data consists of 155 males and 47 females. The age range was from 67 to 75 years. Retrospective data and other patient profiles were also collected. Two experienced neuroradiologists manually traced the lumen area. An ultrasound scanner with a 7.5 MHz linear array transducer was used to perform the scans [67].

Hong Kong database (HKDB): A total of 300 images from 50 subjects, both symptomatic and asymptomatic, were acquired by a 13.5 Mhz linear transducer. Trained observers with over 10 years of experience manually traced the lumen borders using the ImgTracer application. The outputs were marked as a set of traced (x, y) coordinates [67].

London (UK): The London dataset contains 150 symptomatic and 196 asymptomatic patients. The mean age of patients was 69.9 ± 7.8 years, and 39% were female. The St. Mary’s Hospital, Imperial College, London, UK ethical committee also approved this study using an ATL machine (Model HDI-3000, Advanced Technology Laboratories, Seattle, WA, USA) working on a linear broadband width 4–7 MHz transducer for acquisition, having a resolution of 20 pixels/mm resolution [68,69]. In patients with ipsilateral cerebral hemispheric symptoms (amaurosis fugax), transient ischemic attacks (TIA’s), and previous history of stroke were classified as symptomatic, where 38 had amaurosis fugax, 70 TIAs, and 88 strokes.

Lisbon (Portugal): The Lisbon dataset consists of 50 symptomatic and 110 asymptomatic scans with a mean age of 67.5 ± 0.77 years. Focal transitory and neurological symptoms were included in this study [69]. Carotid bifurcation plaque images of a total of 146, 99 patients, of which 75 males and 24 females, were obtained from the Instituto Cardiovascular de Lisboa in Lisbon, Portugal. An HDI5000 Philips machine with an L12-5 scan probe with a 5–12 MHz broadband linear array transducer operating in B-mode was used for image acquisition. The images were normalized, and the plaque area was segmented by a medical practitioner. Spline interpolation was used to smooth the resultant image [68].

Spain [70]: This dataset contains 67 ultrasounds of CCA. The data were acquired using a Philips iU22 Ultrasound System with frequency ranges of 9–3 MHz, 12–5 MHz, and 17–5 MHz. The Radiology Department of Hospital Universitario Virgen de la Arrixaca (Murcia, Spain) was the source of the patient data collection.

Multi-institutional [64]: A multi-institutional database consisting of 365 B-mode longitudinal carotid images. The images consisted of four different institutions from different ethnic backgrounds.

Dataset Savas et al. [71]: This dataset consists of 501 images collected from 153 patients. The device used for ultrasound imaging was the Toshiba Aplio 400 Ultrasound Device. The data were labeled as “IMT: 1” and “IMT: 0” by two doctors who are experts in the Department of Radiology at Ankara Training and Research Hospital.

Riha et al. (transverse) [88]: The dataset consists of 283 transverse view images labeled with the positions and sizes of arteries. As the purpose was to design an evolutionary cascade, 283 positive images and 282 negative images, totaling 565 images, were generated as a training set. A testing dataset was acquired separately where video sequences of a total of 50 from 15 adults were collected through an Ultrasonix device from which 583 test images were extracted from every 20th frame. The Toshiba device was used to image the carotid arteries of two adults and a child. The images collected were a total of 433 test images with artifacts extracted from every 10th frame. This dataset is publicly available.

Azzopardi et al. (transverse and longitudinal) [72]: The dataset was acquired from five subjects whose ages ranged from 25 to 40. The images were 250 transverse and 250 longitudinal view images. The subjects were scanned with an Ultrasonix Sonix RP Ultrasound machine (Analogic Corporation, Peabody, MA, USA) equipped with a 14 MHz L14-5 Linear Probe.

Malta Dataset (transverse) [46]: 15 subjects ages 60–80 years were scanned using Ultrasonix. The Sonix RP US machine (Analogic Corporation, Peabody, MA, USA) was equipped with a 14 MHz L14-5 Linear probe, and 50 transverse US images were acquired from this. Malta’s research ethics committee approved the study. The subjects were placed in a supine position, and a probe was placed on their neck for obtaining carotid artery images.

SPARC: The Stroke Prevention and Atherosclerosis Research Centre (SPARC, London, Canada) and Zhongnan Hospital (Wuhan, China). This dataset consists of 144 patients with risk factors such as hypertension or hyperlipidemia. The device used for imaging in this dataset was the HDI 5000 ultrasound system and an L12-5 probe (Philips/ATL, Cincinnati, OH, USA) at a central frequency of 8.5 MHz [73].

Athens dataset [74]: The dataset consists of 12 symptomatic and 41 asymptomatic patients with carotid atherosclerosis. Symptomatic patients are those with existing and diagnosed conditions and those with a prior stroke incident. A video dataset is also available for 18 patients, 4 symptomatic and 14 asymptomatic. There are 74 videos in total, with 58 asymptomatic and 16 symptomatic cases. The plaque region was cropped for classification.

Japan [75]: 204 patients with an average age of 69 ± 11 years old were subjected to a US scan using the Aplio XV, Aplio XG, and Xario; Toshiba, Inc., Tokyo, Japan, scanner. High resolution, 408 right and left ultrasound common carotid artery images were collected from this experiment. IRB, Toho University, Japan, provided the IRB consent.

Houston dataset [79]: The data were collected from color Doppler examinations. Plaque morphology and hemodynamic changes were captured in different regions of the carotid artery. Two datasets were captured, of which 13,810 were from Philips EPIQ and 15,546 by GE LOGIQ E9 device.

NEFRONA [89]: NEFRONA is a set of B-mode US acquired from a Vivid BT09 device (GE), with 6–13 MHz. It consists of 27 images marked by the CIM regions and their CIMT values. The source of data is Atherotrombotic Diseases Unit Detection Hospital Arnau de Vilanova.

REGICOR3 [89]: This dataset consists of images collected from 2379 subjects from Girona’s Heart Registery. The data are collected from the age range of 35 to 84. Ultrasound longitudinal images were generated from images captured by the Acuson XP128 US system equipped with an L75-10 MHz transducer and a computer program extended frequency (Siemens-Acuson). A total of 4751 CCA images and 3733 Bulb images were generated for the carotid artery.

Table 4 is an overall count of the datasets used in the literature, where the quantity of the data is significant for artificial intelligence approaches.

**Figure 7:**
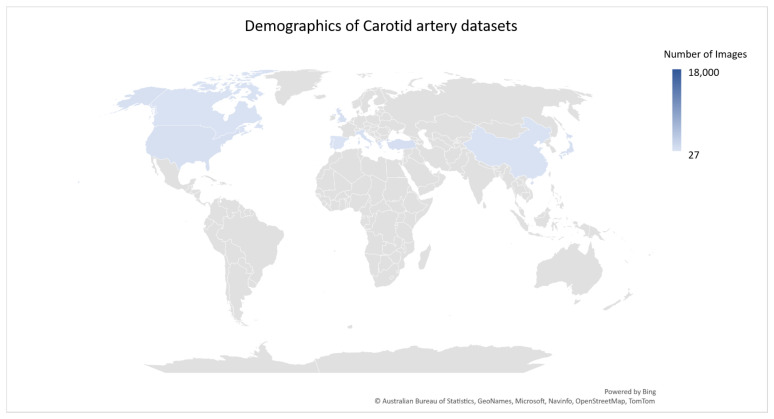
Ultrasound datasets, a demographic representation.

Figure 7 is a geographical distribution of existing datasets across the world. Figure 6 is a representation of the ultrasound image with its manual annotation performed by trained operators.

The datasets are annotated by trained operators, and the dataset validity is tested by correlation studies identifying accuracy, specificity and sensitivity in terms of segmentation tasks and classification accuracy for classification tasks by comparing them to either retrospective data or the radiologist and/or expert observer labels. The standards authority NASCET requires two specialists to annotate and/or score the dataset. Several manual segmentation techniques were utilized in the state of the art for this purpose such as [65], 3D slicer (www.slicer.org (accessed on 2 January 2022)), RectLabel (https://rectlabel.com (accessed on 2 January 2022)), and ITK-SNAP [77]. Inter-observer and intra-observer evaluations with automated and manual methods are used to authenticate the annotation. This is crucial to the localization and diagnosis of the carotid artery.

## 5. Image Analysis

Early image analysis research focused on multi-step filtering and feature extraction methods. Risk stratification of the carotid artery being the main aim of the study, the literature reviews contain domain-wise analysis pertaining to segmentation, localization, and grading, performed individually and as multi-task approaches, where the risk classification is performed in a single pipeline in the state of the art. The datasets available, as reviewed above, are employed in the given state of the art for training and testing.

### 5.1. Machine Learning and Deep Learning

**Figure 8:**
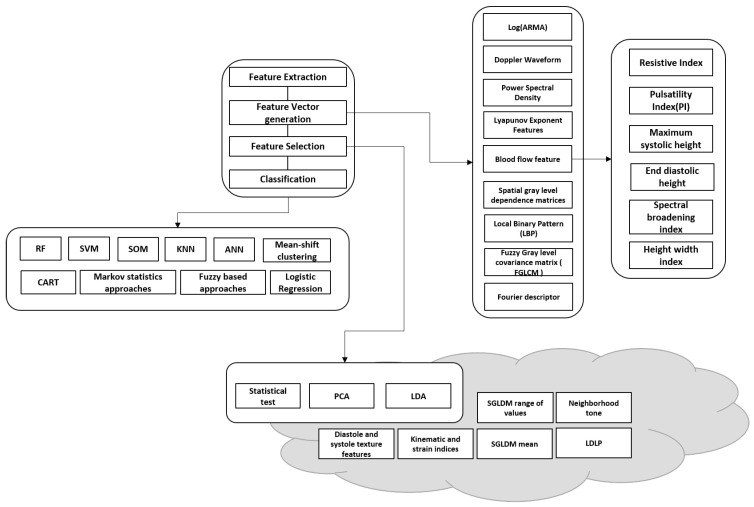
Existing ML methods in literature.

Machine learning and deep learning have become significant for carotid artery image analysis. Figure 8 illustrates existing literature and studies on carotid artery segmentation, grading, and analysis using machine learning.

A typical machine learning pipeline includes feature extraction, feature vector generation, feature selection, and then classification. The features are generated using image processing techniques, and in some cases, retrospective data are also used for correlation. Machine learning systems were developed for plaque wall risk assessment using morphology-based characterization. The fundamental assumption in such systems is the extraction of the gray-scale features of the plaque region. Even though these systems can perform risk stratification, they cannot achieve higher performance due to their inability to select and retain dominant features. The typical flow of the operation involves the detection of the artery (localization in an ultrasound image) and later segmentation of the artery wall/lumen/intima–media/plaque. The artery/lumen/intima–media/plaque is then measured and compared to the gold standard for risk prediction or classification. After training the model on a given dataset, it is evaluated on unseen data to identify the reliability and feasibility of the approach for real-time use. Selecting features that are discriminant and relevant is vital to reducing overfitting and underfitting in ML.

The Atheromatic system was the outcome of a patent [65], which was widely used in several states of the art for carotid artery risk estimation. SVM, KNN, fuzzy classifier, and SOM were widely used as classifiers using Arethromatic 1.0 feature extractors, also called AtheroPoint, Roseville, CA, USA. Blood flow analysis was another technique using non-image data and spectral analysis acquired from Doppler signal, LEAD data, blood pressure and waveform data, and the duplex US was used for carotid artery risk prediction and probabilistic estimation and/or classification [90]. Feature extraction and classification are not a one-step approach to risk prediction. The strongest features are selected by statistical analysis, and classification and prediction are performed for these features accordingly. Performance analysis was orchestrated using statistical tests, covariance, and correlation with the ground truth produced by trained operators, and experts in the field of radiology.

However, in terms of image analysis, the images generated being of low color patterns, that is mostly grayscale, with distinguishing features that may be handcrafted or semi-automated, is one of the downfalls of machine learning [67]. This led to the current evolution of deep learning, where features are fully automatically and statistically extracted using neural networks. Neural networks can identify patterns in data and extract the most relevant features for the task specified based on the annotated dataset. Thus, robust features and efficient classification and prediction are achieved in this process.

Another advantage is that manual feature extraction may be patient-dependent or based on a single expert’s opinion, influencing the outcome of the result. Through deep learning, feature extraction is automated, evaluated based on a chosen loss function, and optimized to obtain the best performance with the best set of discriminating features. Deep learning has another advantage in that it can extract a multitude of hidden features based on the layers in the network. This improves the generalization of the network and discriminates or classifies images efficiently.

Common approaches used in deep learning involve convolutional neural networks (CNN), auto-encoders, transfer learning on pre-trained CNN models, LSTM (long-short term memory), self-attention mechanisms, and transformers, of which CNN and TL widely experiment in the state of the art for this application. Figure is an outlook on how carotid artery stenosis risk classification is performed using CNN-based models with multi-step approaches for segmentation and classification.

**Figure 9:**
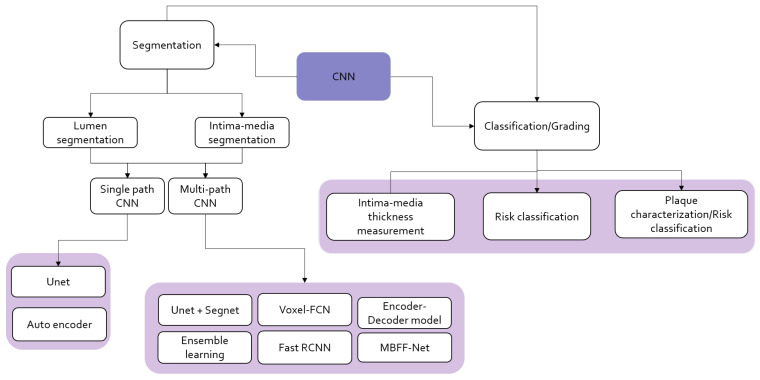
Current methods using CNN.

ML and DL for carotid artery risk estimation perform several tasks that include classification of symptomatic and asymptomatic plaque, risk grading, segmentation of plaque, intima–media and lumen, plaque characterization, and plaque area measurement. The following section will brief on the state of the art related to each main category of application. Figure 8 and Figure 9 are illustrations of existing ML- and CNN-based methods in the literature. Table 5 depicts all the recent existing states of the art in carotid artery risk prediction using machine learning and deep learning from 2015 to the latest.

#### 5.1.1. Classification

Classification is a broad term used to separate given data into two classes. In the state of the art, concerning carotid artery risk prediction for diagnosis, various classes were adopted, as in plaque classification as vulnerable or plaqued [67], or in the classification of symptomatic or asymptomatic in [105]. Those with characteristics of the plaque such as having a bright appearance in the US with the presence of high lipid content and considerably low calcium with a thin fibrous cap are identified as symptomatic, and those with a poor lipid core, dark echoic appearance, and high calcium level are asymptomatic. In addition, quantifying the thickness of the intima–media and classification are based on the gold standard for grading the plaque’s nature. Each element eventually provides information about the status of the carotid artery stenosis.

Table 6 and Table 7 are a description of the recent state of the art in the classification of carotid artery stenosis. The bolded numbers indicate highest performing scores in each metric presented. Most of the state of the art deals with the classification of plaque tissue as symptomatic or asymptomatic. The ML techniques used are mainly trained on extracted features and delineated plaques. They are labeled and classified using SVM [68]. Other techniques by Acharya et al. utilized the wavelet domain for feature extraction. The Fuzzy SVM classifier and SVM with an RBF kernel were the earliest approaches. The maximum accuracy achieved from all the ML approaches, irrespective of the dataset, was 93.1% for the fuzzy SVM classifier using a multi-center dataset [111]. Further, with neural networks, 99.1% accuracy was achieved, but with 54 images [71]. It may question generalization with a few datasets.

In terms of the deep learning approaches, CNN was widely tested on more than 10 algorithms with both fully trained models and transfer learning approaches. Transfer learning utilized existing pre-trained networks for feature extraction or fine-tuning on the carotid artery dataset. The intuition behind the efficiency of CNN entails that it uses the first layers to catch low-level features and, in the later layers, high-level features such as high lipid or calcium content. This can be significant in identifying a symptomatic plaque. Table 6 and Table 7 provide an overall depiction of the CNN architectures used the validation format, the metrics for evaluation, and the result of the evaluation in terms of accuracy, sensitivity, specificity, AUC, and F1-score.

Both supervised and unsupervised approaches were used in the state of the art, including generative adversarial networks for dataset generation in [79], ensemble learning, and transfer learning. Major analysis and ranking of models are performed in [66] to evaluate performance by scoring for the transfer learning models. Data augmentation was used through cross-validation, where 10-fold, 5-fold, 6-fold, and hold out were used in the literature. The metrics vary in the state of the art, but the common ones include, accuracy, AUC, specificity, and sensitivity. In particular, ref. [79] employed the Pareto principle for cross-validation and an 80–10–10 split for generative networks. Two-stage approaches were common, where segmentation was performed first, and then risky and non-risky plaques were classified. In some states of the art, the patented Arethromatic system was utilized to segment and crop the plaque region for classification. The highest accuracy of 97.2% was achieved in [79] marked as bold text in Table 6, which is due to the presence of a generated balanced dataset.

#### 5.1.2. Plaque Tissue Characterization

The graph cut approach, volume estimation using Bayesian techniques inside the plaque region, Rayleigh mixture model for lipids, fibrotic tissue, and calcified plaque identification were the most common algorithmic approaches. Another system achieving 64.96% AUC in the degree of stenosis estimation is Atherorisk. It is a CAD (Computer Aided Design) tool used for identifying plaque echogenicity.

The MFS (Mean feature Strength) was computed by the DCNN11 of [47] where the dataset was augmented 5× for a larger dataset. This defined MFS values for the symptomatic type. In the symptomatic type, MFS was found to be greater for symptomatic plaques, producing a clear demarkation for classification. The bi-spectrum of the plaque falls in the higher order spectra and for symptomatic plaques, it was found to be 54.4% higher than asymptomatic.

Table 8 is the state of the art in plaque characterization, where the plaque was characterized and measured. It is noted that segmentation and classification precede some approaches, and some use existing automated approaches like Arethromatic 1.0 to extract the ROI and then characterize the plaque. In addition, total plaque area measurement was performed using deep learning methods using fully connected CNN, Unet, and Unet++, achieving Δ TPA of 0.73 to 3.91.

#### 5.1.3. Segmentation

Blood vessel segmentation algorithms are the key components of automated radiological diagnostic systems. Segmentation methods vary depending on the imaging modality, application domain, whether the method is automatic or semi-automatic, and other specific factors. Pre-processing and/or post-processing are performed on images to remove the noise characteristics based on the type of noise or artifact embedded. The region of interest is sometimes cropped out and the segmentation of the lumen is performed on the lumen area. The intima–media thickness is then identified for risk stratification and, in some cases, the plaque is characterized. The grading of stenosis is performed as a classification task. A pipeline for segmentation, classification, and risk measurement is achieved in this process.

The challenge comes in the anatomy of the artery, where the intima–media interfaces are close to each other leading to a fuzzified boundary. Distinguishing this boundary is difficult, and the artifacts contained may further cause misrepresentation. The near wall and far wall in the US images may be similar in texture to other tissues, especially in the CCA region, leading to further errors in diagnosis and/or segmentation [113]. Each challenge should be solved during segmentation by providing diverse datasets, pre-processing, and post-processing steps for higher accuracy.

A review of the state of the art in segmentation, classification, and detection for carotid artery stenosis using online automatic detection algorithms includes automatic thresholding, edge-based methods, and morphology-based methods [6]. In particular, the ML methods used for segmentation start with SVM, LDA, and p-jurs algorithms, which are seen in the earliest papers. Furthermore, several states of art include Dynamic Programming Techniques [114], of which are Hough Transform, Nakagami Mixture Modeling, and Active Contour methods, an example of which is identifying the contour of interfaces between the intima layer and between media and adventitia layers, Edge Detection [115], Gradient-Based Techniques; and combined approaches to improve accuracy [4,116]. The algorithms work simultaneously, performing segmentation through dilation, line tracing on the lumen area, vessel diameter, and contouring the intima–media.

Supervised deep-learning approaches based on a dilated U-net network, Unet++, segnet, and Segnet + Unet and Deeplab have been studied by [43,49,67,110] as shown in Table 9.

**Figure 10:**
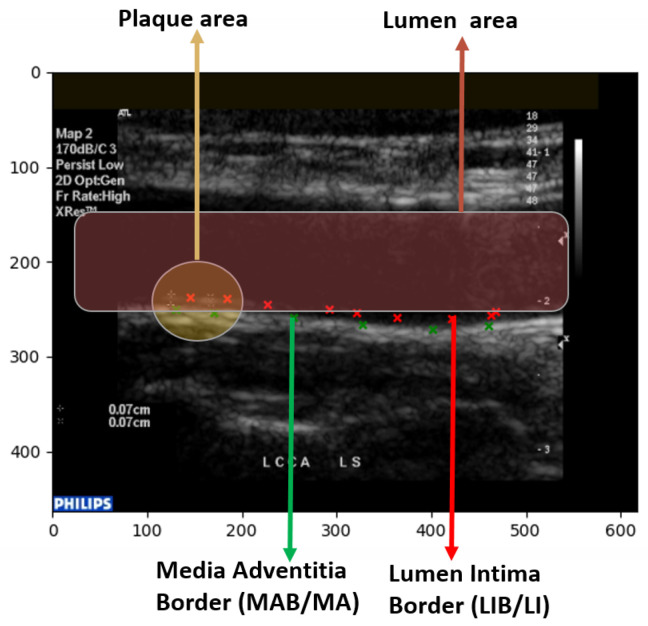
Regions of segmentation and risk analysis.

**Figure 11:**
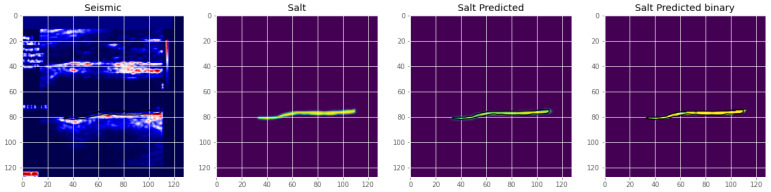
The first US image denotes the original image in the seismic spectrum with clear indication of the lumen area in dark color, the segmented region is bordered by black line. The subsequent images are true masks with the region of interest in green color and the subsequent ones are generated in two different formats [76].

The several regions of segmentation performed on the state of the art are as shown in Figure 10. Figure 11 is an illustration of the Unet segmentation of the carotid artery on the CUBS dataset producing an accuracy of 97.1% and dice loss of 0.93. The plaque is segmented in the predicted mask, which is also represented in binarized format. Salt and seismic charts are represented for visualization and explainability.

Transfer learning approaches have worked well on most of the models for segmentation and have produced higher accuracy. The far wall was detected, and then IMC was segmented from the near wall. A multi-stage approach was used with cross-validation [110]. An inference from the evaluation noted that a failure of the first step, which is far wall detection, will fail the segmentation as a whole, and so the second stage is dependent on the success and accuracy of the first stage. With scale-space and statistical classification combined, a multi-resolution framework was presented with which the lumen–intima and media–adventitia (MA) were segmented. A fuzzy Mamdani-type pre-classifier was used to improve segmentation, which is used as a feature extractor in [8]. The latest state of the art is tabulated in Table 9, where the segmentation approaches are leaning towards deep learning models, specifically, Unet, Segnet, variations of Unet such as dilated Unet, and the encoder–decoder model. Apart from accuracy, sensitivity, and specificity, MCC, Dice similarity, Jacquard Index, and AUC were used as metrics. The Poly-line metric was used in an encoder–decoder model [95]. A modified HD distance was computed in [46,72]. The CIMT error and distance were calculated based on the ground truth in [75,92]. Statistical analysis was performed on each segmentation result, comparing and correlating it with the manual segmentation, which in most cases is the ground truth, implying the importance of accurate annotation.

Transverse image segmentation is dealt with in the state of the art using native 2D images or 3D US image slices. For segmentation, semi-automatic as well as automatic methods have been proposed. Lumen segmentation, media adventitia segmentation with vessel wall volume, and total plaque volume identification and/or segmentation have been a matter of study in recent literature.

Deep learning networks were utilized for transverse view image segmentation in [73], where the segmentation area was the MA and LI interfaces in the US image. An encoder–decoder network modified from the baseline Unet was used to segment the image. Plaque burden quantification requires the LI and MA to be marked efficiently. Other forms of modification on Unet include constrained Unet with a cost function. A stacked convolutional auto-encoder within a TL network was adopted to learn the non-linear compact representation of the anatomy. Similar metrics to US longitudinal methods are used to evaluate the model, such as Dice, IoU, Jacquard Index, etc., mentioned in Table 9.

The segmentation of different regions of the carotid artery is evident in the state-of-the-art, where each dealt with multiple representations of stenosis. The segmentation of the carotid artery is performed, and a mask is generated, and the boundary of the mask is used to estimate several factors such as the lumen–intima boundary (LI), the intima–media thickness (IMT), the media–adventitia (MA) boundary, the lumen thickness, and the plaque area itself. A post-processing step where the thickness through an area of the produced mask is compared to the ground truth uses several performance evaluation metrics that focus on overlap, geometry, and pointwise analysis. In [31], a thorough evaluation of different observer annotations and errors using PDM, MHD, CIMT bias, and point-wise analysis is performed on the segmented image. The lines plotted in Figure 10 represent the lumen–intima (LI) and media–adventitia (MA) boundary in green and red points, respectively. The measure of the thickness is vital to grading the carotid artery stenosis risk.

The lumen area, which is typically 3.5 mm [117], decreases with plaque deposit, and that is taken as a marker for risk stratification. Intima–media, on the other hand, increase in size due to plaque deposits; thus, the larger the size, the higher the risk. Marking the intima–media is performed by identifying the lumen–intima layer and the media–adventitia layer. With the plaque region identified, the amount of plaque can be measured, the plaque can be isolated, and the tissue can be characterized or classified to determine the stenosis level.

## 6. Discussion

Imaging of the carotid artery has progressed from heavy, bulky hardware to lightweight, portable devices equipped with ultrasound systems. Its accessibility has made it a popular tool for assessment, hence its significance in carotid artery stenosis risk assessment.

Analyzing and understanding images were a task for trained and expert annotators, however, with a decade or more of research in automated analysis with conventional multi-step approaches that include thresholding, edge detection, and contour mapping to highly sophisticated deep learning frameworks from which two patents [65,66] have been produced for plaque segmentation and multiple works of literature on each task of computer vision image analysis involving region of interest segmentation, classification, and plaque segmentation, the carotid artery risk stratification using image analysis has been reviewed in its entirety. Further, several algorithms were proposed for risk stratification using distance metrics, plaque characterization, and thickness measurement.

The focus of evaluation shifted to the US as it is the most prominent image-based analysis dataset used due to its ease of use, cost, availability, and the fact that it is the first line of diagnosis for carotid artery risk analysis. Thus, the metrics evaluation and the evaluation of the method were margined on US images in specific B-mode US images.

Analyzing the latest methods used state-of-the-art deep learning and machine learning models for carotid risk stratification include segmentation, plaque characterization, and classification. The requirement to outperform the existing models in this domain to achieve better automated analysis requires an overview of approaches that have been dealt with.

In terms of the datasets available, the majority of the datasets are B-mode ultrasound images. As can be seen in Figure 10, the datasets vary in size, demographics, and use. There are a few publicly available datasets, such as those used in [31,78]. The maximum number of images available are in [31]. Most datasets require a pre-processing step for better performance.

The most recent deep learning methods are primarily based on transfer learning methods for classification and the modification of Unet for segmentation. The segmentation of the intima–media and lumen areas is the most prominent area of research in the state of the art, as seen in Table 9. Cropping the region of interest enables better classification of the plaque, and thus accurate segmentation is essential for a reliable diagnosis. Rather than using extensive feature extraction techniques, deep learning methods display better accuracy through the discovery of hidden discriminative features using a loss function.

The metrics used for deep learning models for classification and segmentation not only delve into the pixel-based accuracy but also customize the loss function based on dice loss and binary cross entropy loss for segmentation.

**Figure 12:**
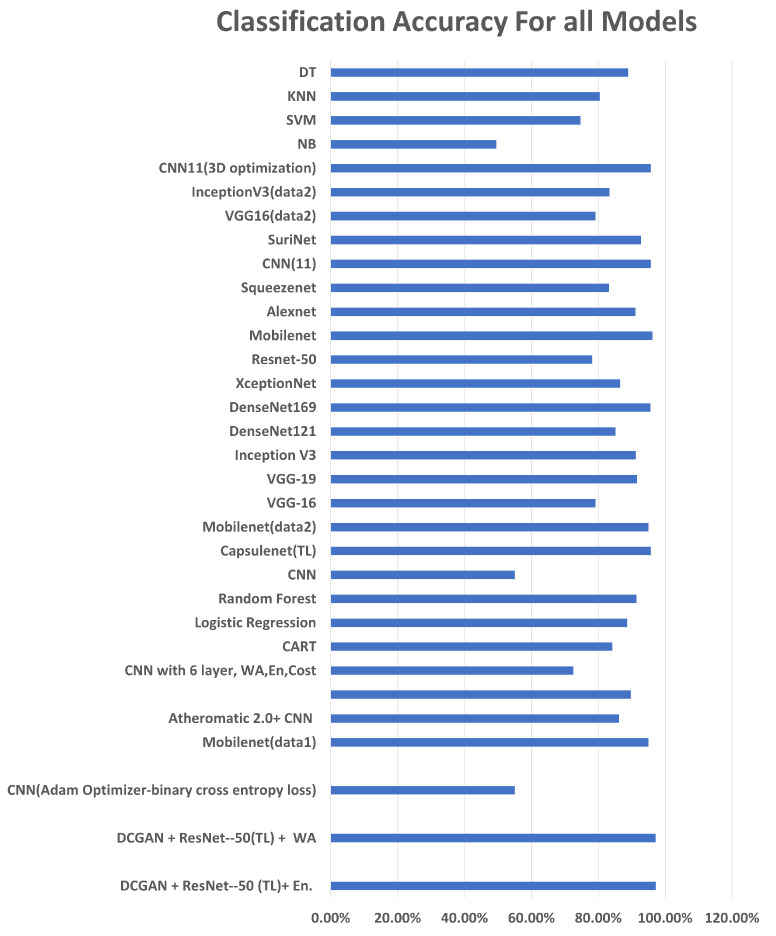
Comparison of accuracy of classification using deep learning. The task performed may vary in terms of dataset, annotation and the region of interest.

Classification problems characterized the region and/or plaque into categories such as normal and abnormal, risky and non-risky, and computed the total plaque area for measuring the plaque thickness, leading to risk stratification. DL models, specifically TL, have been extensively used in two consecutive papers by Sanagala et al. [47] and Saba et al. [67]. Through transfer learning, the complexity of training is decreased. However, accuracy may be at stake as the best-performing model using an ensemble of approaches by Lindsey et al. [79]. A generative network (DCGAN) was used to create a dataset for the imbalanced class and was trained with Resnet-50, a pre-trained CNN model. Ensemble learning was performed with a weighted average and cost function. This shows that enhanced feature descriptors perform better. However, the margin of tolerance is low, so it can be considered a comparable performance to Mobilenet, which has approximately 1% lower accuracy than Lindsey et al. [79], as seen in Figure 12. The datasets used and the quantity and quality of images in each vary for both. This could be a contributing factor to the outstanding performance.

Further, referring to Table 7, we see the highest results marked as bold for the OSPF-network in [105] in terms of sensitivity, specificity and F1-score from those that are available. The significance of a hybrid view of images is used in this training, wherein the plaque region was extracted from transverse as well as longitudinal images of the carotid artery. The model consists of four feature extraction networks that extract features from the data, which were segmented manually. The complex four-layer network with a hybrid dataset supported high classification accuracy. However, in real-time use cases, latency needs to be experimented with. In addition, a correlational study is required to be performed with retrospective data with regards to the accuracy of classification or a final say on whether the person is stenotic or at risk. The progression of the disease can be further identified with data collected at different times.

Segmentation approaches reigned as the state of the art in carotid artery risk stratification being the initial step in most classification approaches, which requires a region of interest drawn out for efficient categorization. The segmentation models are mainly based on an encoder–decoder model, standardized as Unet. Unet is a convolutional neural network designed for biomedical image segmentation and works with fewer images. The factor that enables Unet to perform better in segmentation tasks is its ability to localize and classify the pixels. A standardized metric is not present in the state of the art. Segmentation involves segmenting the lumen diameter, plaque area, and intima–media layer and then later measuring the intima–media thickness, lumen–intima boundary, and media–adventitia boundary, which were markers for identifying the performance of the model. The dice similarity coefficient was used to identify the accuracy of lumen segmentation, and the intima–media–adventitia layer. The distance between the lumen–intima and intima–media was further compared with expert manual segmentation, where benchmarking was performed based on different distance metrics, euclidean distance, polyline distance, and modified Hausdorff distance, as can be seen in Table 9 where the greatly metrics differ. Meiberger et al. [76] presented a standardized approach with their dataset as polyline distance metric (PDM) and MHD.

**Figure 13:**
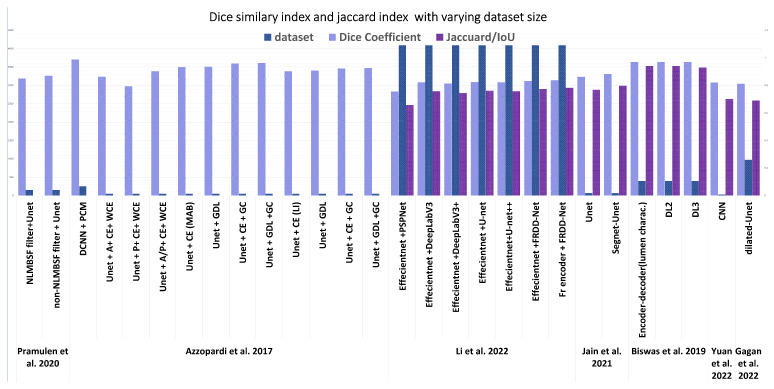
Segmentation benchmarking using dice similarity coefficient and Jaccard index as mentioned in latest state of the art (Azzopardi et al. 2017 [72], Pramulen et al. 2020 [44], Li et al. 2022 [118], Jain et al. 2021 [103], Biswas et al. 2019 [109], Yuan et al. 2022 [77], and Gagan et al. 2022 [78]). The type of dataset and the region of segmentation varies in each model [44,72].

Figure 13, indicates the number of images and the dice coefficient of several significant recent methods after segmentation. The latest in segmentation in [113] performs the segmentation using reinforcement learning; however, the metric used is in terms of mean absolute error that measures the euclidean distance as 60 μm, which is compared with [76], which was computed as 114 μm. However, the dataset size and origin vary enough for a fair comparison. Three-dimensional US datasets were compared for dice similarity of the latest in [113], which benchmarked on a dice similarity coefficient of 91.9% accuracy using Voxel-FCN. In conclusion, for segmentation, the dataset origin, size, region of interest, and metric used vary significantly in each method.

**Figure 14:**
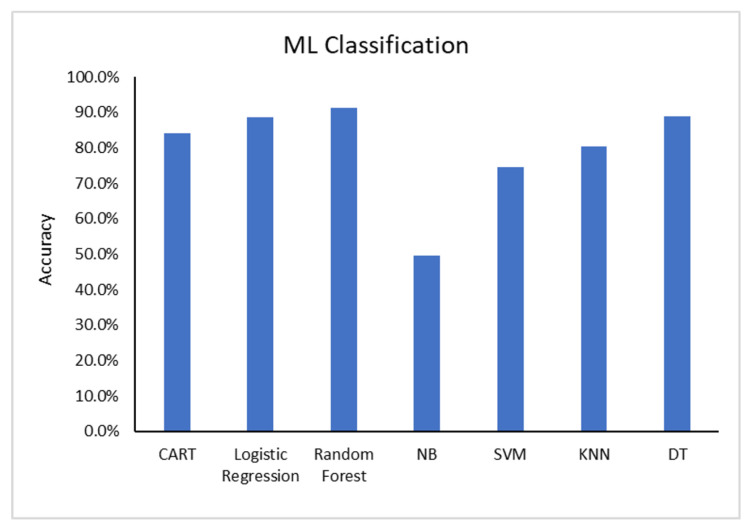
Comparison of accuracy of classification using machine learning techniques. The task performed may vary in terms of dataset, annotation, region of interest, and features selected.

As can be seen in Figure 14, a comparison of the latest models in the literature, the Random Forest classifier achieved the highest accuracy.

**Figure 15:**
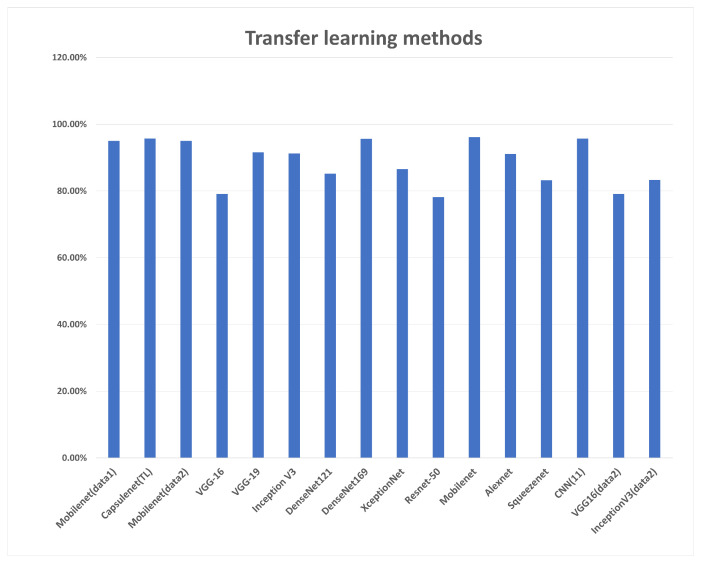
Accuracy of classification using deep-transfer learning methods.

In terms of transfer learning methods charted in Figure 15, all models presented comparable performance on two different datasets, where mobilenet achieved the highest accuracy. Segmentation was the most evaluated approach for a carotid artery, with the latest papers focusing on segmenting the intima–media and estimating the plaque thickness. Table 9 describes most of the approaches for this task. The evaluation metrics used are varied, as some of them evaluate using common segmentation metrics such as Dice loss and Jacquard index, while others evaluate the mask generated task-wise such as LI measurement. The accuracy of segmentation is essential to risk stratification as it defines the area of the plaque. From the segmentation approaches used, the convolutional neural network Unet and its modifications were extensively used for this approach, and the architecture is modified in the literature to achieve better results. Its encoder–decoder model and its design for small datasets have their relevance stated in different modifications of it used for segmentation in not just this domain, but several bio-medical segmentation domains.

Inferring from the approaches and datasets used, there is a requirement to evaluate the models using standardized datasets for better comparison of the models. Medical datasets are a privacy concern and need to undergo rigorous IRB (Institutional Review Board) approval schemes for the sake of ethical research, which adversely affects their availability, where most state-of-the-art research has not publicly shared the dataset used. Further, the quality of the image is a deciding factor in performance, thus several pre-processing techniques were proposed, as seen in Section 5.

## 7. Challenges

There are many challenges yet to be addressed in carotid artery risk stenosis that include dataset availability, algorithms for real-time use cases, tracking stenosis using retrospective data, image artifacts that may arise due to equipment, annotation errors in labeling data, and automated annotation, among a few.

In particular, the intima–media thickness has been defined as the metric to estimate the stenosis in the carotid artery. The artery may contain regions that are thin and may merge with the endothelial tissues beneath the intima. This leads to a misidentification of the intima–media line.

The datasets used in the state of the art mostly do not allow public access. For further research and benchmarking, their availability would be advantageous. There are many limitations to the dataset noted in this study, with image quality being a major concern with a plethora of artifacts found in the state of the art based on device and motion [119]. The datasets are from certain regions, as depicted in Figure 7, leading to bias. The number of images for analysis does not exceed 300 in most datasets where deep learning methods require more data.

Further, low latency is a requirement in medical applications, and thus the use of multi-shot approaches increases the latency, delaying a real-time use case. In some states of the art, the region of interest is cropped manually, referred to as a semi-automatic approach, which adds to the time complexity.

Although DL methods provide significant accuracy in classification, the computation time and memory usage are high compared with traditional image processing and machine learning. Furthermore, multiple iterations and hyper-parameter tuning are required to achieve the best model. With small datasets, generalization is a major challenge; data may overfit to that particular trained dataset. This requires further techniques such as max pooling, drop-out layers in the deep learning model, or multiple iterations to retrain the model. Data augmentation is a method to improve dataset size; however, generalization in terms of the source is a challenge in this approach.

## 8. Future Directions

- Going beyond stenosis: Measuring arteries may provide insufficient information about plaque burden. Further analysis of 3D images can improve plaque diagnosis in its initial stages. Other factors, like lumen ulceration and diseases affecting this part of the carotid artery, should be evaluated. Using advanced algorithms for analysis, such as vision transformers and attention-based methods for segmentation, can be future research as more features can be identified through these methods.
- TinyML for deploying the algorithms on Internet of Things (IoT) devices for self-diagnosis or patient-doctor communication. Compression, quantization, and optimization of the models still need to be researched for this purpose. The trade-off in terms of accuracy should be intolerable for medical applications. Online learning can be implemented on devices and this can be beneficial for lightweight applications.
- Apart from image-based analysis, video segmentation and tracking are approaches that require further study. A transverse dataset in [120] was utilized for this approach. As the artery shifts due to movement tracking, the artery will enhance diagnosis and capture its dynamics while in motion.
- A multicultural, unbiased dataset is required across multiple institutions across continents for carotid artery stenosis to develop a generalized carotid risk prediction model. The quality of datasets is a determining factor for good performance. Therefore, there is a requirement for a high-resolution dataset for analysis.
- Correlation studies with other diseases can be analyzed using retrospective data related to patient profiles and existing health conditions.
- Other imaging modalities have been proposed, such as infra-red thermography [121], active dynamic thermography [122], and skin temperature maps by Saxena et al. [121]. Skin temperature maps of the carotid artery of the normal subjects had a higher average temperature. These maps can further improve diagnosis with other imaging modalities for a better and more accurate diagnosis.
- Most datasets were taken at one point in time, before or after a stroke. However, progression of the disease or improvement can be charted if the images were taken multiple times from diagnosis to treatment. Further finding correlations and convergence in the data.

## 9. Conclusions

With an in-depth examination of carotid artery stenosis risk stratification, the most common methods are reviewed in terms of imaging modalities, the tools used, and the techniques for automated image analysis. The performance metrics of automated tools are analyzed, and models with similar metrics are compared. The best-performing model in terms of deep learning has been identified. Plaque segmentation, classification, plaque characterization, and risk quantification based on grading the plaque are automated through several techniques, including machine learning and deep learning. It is noteworthy that deep learning models produce significantly higher results than ML models. Despite the increased complexity, deep learning models efficiently extract classifying features of plaque. The main challenge in automated analysis using deep learning and machine learning is the requirement of large datasets with a substantial number of publicly available images for testing models.

## Figures and Tables

**Table 1:** Carotid artery stenosis grading baseline.

Degree of Stenosis	ICA-PSV (m/s)	Plaque Est.	ICA/CCA-PSV Ratio	ICA EDV (m/s)
Normal	<125	None	<2	<40
<50	<125	<50	<2	<40
50–69	125–230	>50	>4	<135
>70	>230	>50	>4	>135

**Table 2:** Correlation evidence for CTA, MRI, and US for carotid artery stenosis risk analysis.

Ref.	Method	Data	Sensitivity	Specificity	Accuracy	Correlation Data
[28]	Ultrasound arteriography	transverse cross view images	100	85	NA	Vessel X-ray
[32]	CTA imaging	Vessel images	NA	NA	82 Agree	Expert observer
[33]	CTA and micro-CT	50 arteries in 27 patients	NA	NA	72.6 Agree	4 Expert observers
[19]	Doppler-Invitro	Blood flow analysis	NA	NA	NA	NA
[25]	Thoracic sounds	Audio signal	NA	NA	NA	NA
[27]	Speckle in Doppler	Spectral signal	NA	NA	NA	Chi-squared test
[34]	Doppler Ultrasound	Spectral signal	NA	NA	94–100	NA
[2]	MRI	Vessel Images	92.6	82.7	NA	DSA images

**Table 3:** Image enhancement in recent studies.

Reference	Image Pre-Processing Techniques	Type of US
[44]	De-speckle using NLMBSF [45]	B-mode(Transverse)
[46]	Bimodal fusion of amplitude and phase congruency	B-mode
[47]	Grey scale median, fractal dimension B-mode higher order visual heatmaps.	B-mode
[48]	Edge detection using Canny and Sobel	B-mode
[49]	Curvelet decomposition	B-mode
[50]	CLAHE (Contrast Limited Adaptive Histogram Equalization)	B-mode (Transverse)

**Table 4:** US Datasets in State-of-the-art.

Name	Number of Images
Japan dataset [67]	404 (private)
HKDB(HongKong) [67]	300 (private)
London(UK) [68,69]	346 (private)
Lisbon(Portugal) [68,69]	140 (private)
Spain [70]	67 (private)
Multi-institutional [64]	365 (private)
[71]	501 (private)
[72]	500 (private)
[46]	81,000 (augmented/Private)
SPARC [73]	144 (private)
Athens [74]	74 (private)
Japan2 [75]	408 (private)
CUBS2 [76]	2176 (public)
CUBS1 [31]	500 (public)
CULEX [43]	135 (private)
[77]	30 (private)
SPLab [78]	197 (long.) & 84 (Trans.)
Houston [79]	29,356 (Doppler US-private)

**Table 5:** DL/ML in carotid artery risk prediction (US).

Method	Dataset Size	Task	ML/DL Techniques
[67]	London,	Segmentation + Classification	TL using Atheromatic 2.0
[46]	81,000 US images (Aug)	Classification	Networks stochastic gradient
[91]	45 US images	Segmentation	Auto-Encoders + Deep learning
[92]	67 US images	Segmentation + Classification	Auto-encoders
[93]	15 patients (IVUS and B-mode)	Feature extraction + classification	SVM
[94]	90,000 US patches		CNN model
[95]	396 B-US-203 patient	Segmentation	auto-encoder
[71]	501 US-153 patients	classification	Deep learning
[96]	302 images	Segmentation of lumen	Single Unet and dual path-Unet
[79]		Classification	Resnet
[97]		Segmentation	SuriNet, DCNN
[98]	433 images	plaque localization	FRCNN
[44]	Dataset with NLMBSF and	Segmentation	Unet
[99]	92 Images (aug-500)	Classification	CNN/MobileNet
[100]	10 images	Localization	based on block matching
[101]	1007 3D US images.	Segmentation	Voxel-FCN
[66]	346 patients (Aug6x.)	Classification	TLNN, DNN
[102]	970 ICA from 99 high to risk patients.	Region Segmentation	Unet, Unet+, Segnet + Unet, Segnet-Unet++
[47]	200 + 270 images	Classification	Transfer learning, Densenet, Mobilenet, Alexnet, Squeezenet, XceptionNet, ResNet50
[103]	330 Japanese and 300 Hong Kong	Segmentation	UNet
[104]	self-collected.	Localization and Segmentation	Fast RCNN, Alexnet
[48]		Segmentation	Encoder decoder model with multistage input.
[49]	202 normal-159 abnormal	Classification	CART decision tree, CNN, MobileNet, CapsuleNet
[70]	67 US images	Segmentation	Unet
[105]	1332 US images (Hybrid)	Classification	OSPF-Net
[106]	SPARC	Segmentation	UNet++
[72]	250 Trans., 250 long.	Segmentation	DCNN
[31]	500 images	Segmentation and IMT	dynamic programming, deformable models, Gaussian derivative filters, Unet
[107]	82 subjects	Segmentation	Elastic modulus is measured using DNN.
[108]	510 plaques from 144 patients	Segmentation and TPA measurement	Two UNET models
[91]	55 images of CCA	Segmentation	Autoencoders and Deep learning
[75]	408 left and right CCA	Segmentation and Characterization	2 Stage Deep learning
[109]	408 left and right CCA	Segmentation & Characterization	CNN, FCN, Encoder-decoder
[50]	257 trans.	Segmentation	Unet
[78]	197 long. and 84 trans.	Segmentation	dilated-Unet
[77]	30 (Aug applied)	Segmentation	CNN
[110]	2716 images (CUBS)	Segmentation	dilated-Unet

**Table 6:** Performance of state-of-the-art classification methods of CCA/ICA risk based on accuracy.

Method	Architecture	Accuracy (%)
[79]	DCGAN + ResNet-50 + En.1	**97.20**
	DCGAN + ResNet-50 + En2	97.13
[99]	CNN(Adam Opt.BCE)	55.00
	Mobilenet(TL)	95.00
[66]	Atheromatic 2.0+ CNN	86.17
[49]	CART	84.21
	Random Forest	91.41
	Capsulenet(TL)	95.70
[47]	VGG-16	79.12
	VGG-19	91.56
	Inception V3	91.24
	DenseNet121	85.17
	DenseNet169	95.64
	XceptionNet	86.55
	Resnet-50	78.20
	Mobilenet	96.19
[66]	VGG16	79.11
	InceptionV3	83.33
	CNN11(3D optimization)	95.66
	NB	49.50
	SVM	74.65
	KNN	80.44
	DT	88.90

**Table 7:** State of the art in the classification of CCA/ICA risk.

Method	Architecture	Sensitivity	Specificity	AUC	F1-Score
[74]	CNN6, WA, En.&Cost	75 ± 17.6%	70 ± 10.3%	73 ± 10.7%	
[49]	CART	88.72%	78.34%	83.53%	81.19%
	Logistic Regression	93.46%	81.63%	87.55%	85.41%
	Random Forest	96.11%	85.61%	90.63%	89.49%
[105]	VGG16	81.8 ± 5.0	88.0 ± 3.3		79.8 ± 5.4
	FP-VGG16	88.4 ± 6.8	97.3 ± 3.7	-	91.2 ± 4.7
	OSPF-net	**96.2 ± 3.7**	**97.6 ± 2.0**		**95.9 ± 2.8**
[47]	VGG-16	-	-	0.791 (*p* < 0.0001)	
	VGG-19	-	-	0.915 (*p* < 0.0001)	
	Inception V3	-	-	0.912 (*p* < 0.0001)	
	DenseNet121	-	-	0.851 (*p* < 0.0001)	
	DenseNet169	-	-	**0.956** (*p* < 0.0001)	
	XceptionNet	-	-	0.851 (*p* < 0.0001)	
	Resnet-50	-	-	0.780 (*p* < 0.0001)	
[66]	VGG16			0.791 (*p* < 0.0001)	
	InceptionV3			0.833 (*p* < 0.0001)	
	CNN11 (3D optimization)			**0.956 (** ***p*** **< 0.0001)**	

**Table 8:** The State of the art in plaque characterization and measurement.

Method [Year]	Method	Details	Train-Test-Split	Metric	Result
[112]	Plaque Characterisation	Mean strength of feature map (MFS),	K10	Accuracy	86.17%
		Mandelbrots fractal dimensions		AUC	0.86 (*p*-value < 0.0001)
[66]	Plaque Characterisation	Mean feature strength,	K-10	Accuracy	95.66
		Bi-spectrum, Histogram		AUC	0.956 (*p*-value < 0.0001)
[75]	Plaque Characterisation	2-stage DCNN	K10	CIMT error	0.093 ± 0.0637 mm.
[95]	TPA measurement	CNN + FCN	K10	Δ TPA	2.7393 ± 2.3702
[73]	TPA measurement	Unet	33, 34, 33–44	Δ TPA	0.73 ± 9.63
		Unet++		Δ TPA	3.91 ± 9.46

**Table 9:** State-of-the-art for LI/MA/IMT/LD/Plaque segmentation.

Ref.	Arch.	Metric	Result
[44]	NLMBSF filter+Unet	Accuracy	97.60%
		Dice	85.50%
	non-NLMBSF filter + Unet	Accuracy	97.74%
		Dice	87.22%
[72]	DCNN + PCM	Dice	98.8%
		MHD	0.050 mm
[46]	250	Dice	88.1%
		MHD	5.011
[75]	2 stage DCNN	CIMT error	0.0935 ± 0.0637 mm.
[92]	Extreme learning-AE	CIMT distance	5.79 ± 34.42 mm
[70]	Unet	Accuracy	98.97% ± 0.82
		Dice	86.16% ± 9.98
		Jaccard	76.72% ± 12.10
	Segnet-Unet	Accuracy	99.08% ± 0.66
		Dice	88.23% ± 7.75
		Jaccard	79.63% ± 10.29
[95]	Encoder–decoder	Jaccard	0.94
		Dice	97%
		AUC	0.95
		PDM	99.61%
	DL2	Jaccard Index	0.94
		Dice	97%
		AUC	0.91
		PDM	97.75%
	DL3	Jaccard Index	0.93
		Dice	97%
		AUC	0.93
		PDM	99.89%
[110]	Dilated-Unet	HD LI dist	119 ± 124
		HD MA dist	107 ± 120
		IMT dist	161 ± 159
[77]	CNN	Dice	0.820 ± 0.066
		Jaccard	0.701 ± 0.094
		MHD	1.43 ± 1.27
[78]	dilated-Unet	Dice	0.812 ± 0.068
		Jaccard	0.690 ± 0.095
		Accuracy	0.969 ± 0.014
		MHD	1.71 ± 1.37
[50]	Unet	Dice wall	94.91%
		IoU wall	84.72%
		Dice Lumen	94.22%
		IoU Lumen	77.90%
	Segnet	Dice wall	81.23%
		IoU wall	74.12%
		Dice Lumen	79.11%
		IoU Lumen	70.08%
[113]	Multi-agent RL	MAE(pixel/mm)	3.02 ± 2.23/0.18 ± 0.13 (LD)
		MAE(pixel/mm)	0.96 ± 0.70/0.06 ± 0.04 (IMT)

## Data Availability

Not applicable.
